# Home Treatment for Acute Mental Health Care: Protocol for the Financial Outputs, Risks, Efficacy, Satisfaction Index and Gatekeeping of Home Treatment (FORESIGHT) Study

**DOI:** 10.2196/28191

**Published:** 2021-11-09

**Authors:** Sara Levati, Zefiro Mellacqua, Maria Caiata-Zufferey, Emiliano Soldini, Emiliano Albanese, Maddalena Alippi, Emilio Bolla, Raffaella Ada Colombo, Severino Cordasco, Wolfram Kawohl, Giuseppina Larghi, Angela Lisi, Mario Lucchini, Simona Rossa, Rafael Traber, Luca Crivelli

**Affiliations:** 1 Competence Centre for Healthcare Practices and Policies Department of Business Economics, Health and Social Care University of Applied Sciences and Arts of Southern Switzerland Manno Switzerland; 2 Organizzazione sociopsichiatrica cantonale Mendrisio Switzerland; 3 Research Methodology Competence Centre Department of Business Economics, Health and Social Care University of Applied Sciences and Arts of Southern Switzerland Manno Switzerland; 4 Institute of Public Health Faculty of Biomedical Sciences Università della Svizzera Italiana Lugano Switzerland; 5 Psychiatric Services Aargau Argau Switzerland; 6 Department of Sociology and Social Research University of Milan Bicocca Milan Italy

**Keywords:** acute mental healthcare, home treatment, crisis resolution, home visits, mental health, home care, crisis, home, community-based, mental health services, economic, risk, risks, efficacy, public health, accessibility

## Abstract

**Background:**

Crisis Resolution and Home Treatment (CRHT) teams represent a community-based mental health service offering a valid alternative to hospitalization. CRHT teams have been widely implemented in various mental health systems worldwide, and their goal is to provide care for people with severe acute mental disorders who would be considered for admission to acute psychiatric wards. The evaluation of several home-treatment experiences shows promising results; however, it remains unclear which specific elements and characteristics of CRHT are more effective and acceptable.

**Objective:**

This study aims to assess the acceptability, effectiveness, and cost-effectiveness of a new CRHT intervention in Ticino, Southern Switzerland.

**Methods:**

This study includes an interventional, nonrandomized, quasi-experimental study combined with a qualitative study and an economic evaluation to be conducted over a 48-month period. The quasi-experimental evaluation involves two groups: patients in the northern area of the region who were offered the CRHT service (ie, intervention group) and patients in the southern area of the region who received care as usual (ie, control group). Individual interviews will be conducted with patients receiving the home treatment intervention and their family members. CRHT members will also be asked to participate in a focus group. The economic evaluation will include a cost-effectiveness analysis.

**Results:**

The project is funded by the Swiss National Science Foundation as part of the National Research Program NRP74 for a period of 48 months starting from January 2017. As of October 2021, data for the nonrandomized, quasi-experimental study and the qualitative study have been collected, and the results are expected to be published by the end of the year. Data are currently being collected for the economic evaluation.

**Conclusions:**

Compared to other Swiss CRHT experiences, the CRHT intervention in Ticino represents a unique case, as the introduction of the service is backed by the closing of one of its acute wards. The proposed study will address several areas where there are evidence gaps or contradictory findings relating to the home treatment of acute mental crisis. Findings from this study will allow local services to improve their effectiveness in a challenging domain of public health and contribute to improving access to more effective care for people with severe mental disorders.

**Trial Registration:**

ISRCTN registry ISRCTN38472626; https://www.isrctn.com/ISRCTN38472626

**International Registered Report Identifier (IRRID):**

DERR1-10.2196/28191

## Introduction

Over the last three decades, mental health care in many Western societies has been characterized by a strong emphasis on the sociopsychiatric approach [1]. This has contributed to a radical process of deinstitutionalization (ie, the decline in the number of beds) and transinstitutionalization (ie, an increase in the number of mental health beds in general hospitals and nursing homes) through the establishment of patient- and community-focused mental health care services [2-4]. This shift represents a move away from a system in which patients’ needs were determined and met by health systems toward a “nothing about me without me” system, in which patients’ self-determination, as well as service users’ and carers’ experience of care are considered fundamental for the success of the service provided. Crisis Resolution and Home Treatment (CRHT) teams are one of several types of community-based mental health services offering valid alternatives to hospitalization [1]. CRHT teams take care of people with severe acute mental disorders that would be considered for admission to acute psychiatric wards. Their main tasks include assessing people during a mental health crisis, providing intensive support, and developing a treatment plan to deliver ad hoc services in the patient’s home, on a daily basis, until the crisis is resolved or until the patient is stabilized and can be transferred to community services or private psychiatrists for further long-term care. The interventions of CRHT teams are therefore restricted to acute crises and should not exceed the length of an otherwise indicated hospital stay (typically no longer than 1 month).

The evaluation of home-treatment experiences shows promising results. Since the 1960s, several studies, including randomized controlled trials (RCTs) [5-15] and nonrandomized comparative studies [16-19], have explored the feasibility of managing psychiatric crises at home rather than in hospitals. The extensive literature reviews conducted by Johnson [1], Joy [20], and Burns [21] highlight that: (1) all the studies investigating outcomes from precursors of CRHT demonstrated a reduction in admission rates when home care was available; (2) findings on symptom severity and social outcomes have been more heterogeneous, although they generally favored the home-treatment group in cases where significant differences were reported; (3) improvements in community-based mental health teams may lead to greater benefits for patients; and (4) some concerns and uncertainties exist about the validity of the evidence owing to some marked differences between the groups recruited in most studies, in terms of gender, diagnosis, and housing. Further observational studies also demonstrated a reduction in readmission rates and a decline in bed occupancy following the introduction of CRHT [22,23]. Other studies suggested an overall impact of CRHT in reducing voluntary admissions, whereas evidence of the impact of CRHT on compulsory admissions is still limited and requires further investigation [24,25]. Overall, the inclusion of a psychiatrist within the CRHT team and the provision of 24-hour service appeared to be beneficial; however, it remains unclear which specific elements and characteristics of CRHT are more effective and acceptable, and whether they are equally effective across patient groups [26]. The cost-effectiveness of home treatment compared to inpatient services has never been formally investigated. Informed decisions by policymakers and relevant stakeholders are currently hampered by the paucity of studies on relevant aspects of the CRHT, which is probably also explained by the practical and ethical difficulties of conducting research in the area of mental health crisis.

In terms of acceptability among users, only a small number of studies have investigated patients’ and carers’ opinions in relation to CRHT services. Nolan [27] explored users’ and carers’ perspectives on the use of alternative services and found that most patients had positive views about being treated at home rather than in hospital, and similar outcomes were identified by Hopkins and Niemiec [28]. However, potential drawbacks of CRHT include difficulties in dealing with several health and social care professionals, discontinuity of care between CRHT and community mental health care, and a perceived excessive treatment focus on medication compliance. As for the impact of CRHT on carers, most of the limited evidence dates back to the 1980s [29] and the early 1990s [30]. A more recent survey found that up to 55 percent of carers expressed a preference for home treatment over hospital care [31]. However, the authors suggested that a longer history of repeated acute episodes and limited familiarity with innovative CRHT might have influenced their observations.

In the last 15 years, several CRHT services have been implemented and tested in Switzerland. In August 2007, for example, the Canton of Lucerne developed the first CRHT Swiss service in response to a severe shortage of psychiatric beds. Findings indicated the feasibility of the service and highlighted its acceptability by patients and families, as well as its economic sustainability [32]. In more recent years, the Canton of Aargau, the Canton of Zürich, and the Canton of Ticino have launched their independent CRHT services [33,34]. Compared to other Swiss CRHT experiences, Ticino represents a unique case, wherein the implementation of a CRHT team is backed by the closing of one of the acute wards at the Cantonal Psychiatric Clinic (CPC), a public psychiatric hospital located in Mendrisio, Switzerland. The service design and its evaluation are rooted in the British home treatment experiences [1]. Moreover, a pilot conducted in late spring 2016 explored the feasibility of conducting a mixed methods study in order to formally evaluate the intervention [35]. This study aims to assess the clinical efficacy of CRHT in Ticino; explore its determinants of feasibility and acceptability; and evaluate the cost-effectiveness of CRHT as an alternative to hospitalization to treat acute crisis for people affected by severe mental health disorders.

## Methods

### Study Design

**Figure 1:**
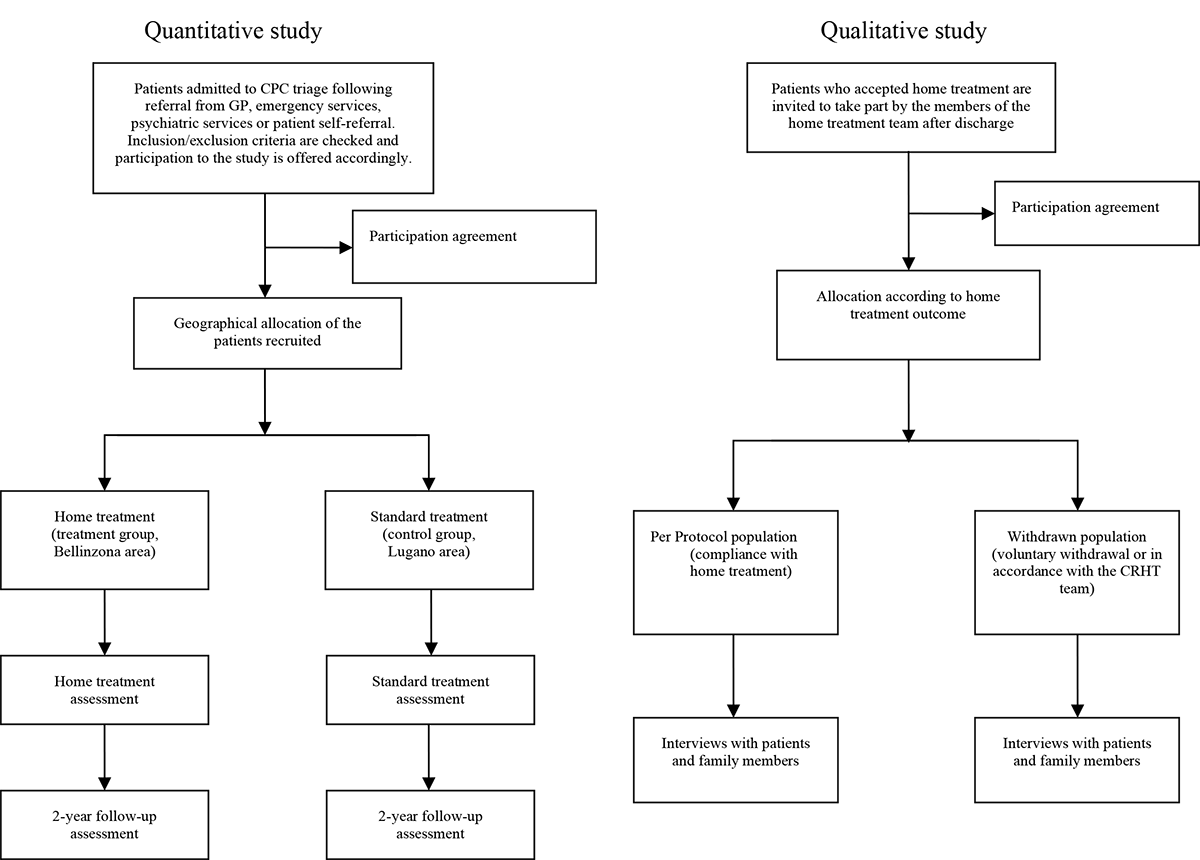
Flowchart of the study design. CPC: Cantonal Psychiatric Clinic; CRHT: Crisis Resolution and Home Treatment.

This study adopts a mixed methods approach, which includes a quasi-experimental design and a qualitative study over a 48-month period (see Figure 1). The qualitative and quantitative approaches are adopted to evaluate the CRHT service from multiple perspectives, including its cost-effectiveness. The study has been registered as an interventional, nonrandomized, quasi-experimental study (registration number ISRCTN38472626).

Quasi-experimental study designs allow the research team to control the treatment, but they do not include random assignment of participants [36]. In addition, quasi-experimental study designs use existing or predefined groups; thus, they are often more convenient and less disruptive than a true experimental design. In the proposed study, in particular, the recruitment process typical of an RCT would pose practical and ethical issues. From a practical perspective, the inclusion of potential patients outside the Bellinzona area, the northern area of the Canton (where the CRHT team is based) in the intervention group would significantly extend the area to be covered by the new home service. Consequently, this would pose major logistic issues that could affect the implementation of the CRHT intervention. From an ethical perspective, the random allocation process of an RCT would leave potential patients affected by acute mental disorders without the possibility to choose between a home-based treatment intervention and hospitalization. A quasi-experimental study design, on the other hand, allows the research team to demonstrate causality between the proposed intervention and a series of predefined outcomes without having to control the random assignment of participants.

### Study Setting

The new intervention is being implemented in the Canton of Ticino, which has approximately 350,000 inhabitants and is located in Southern Switzerland. Acute mental health crises are usually managed by one public hospital and three private clinics. Three of these structures (ie, the public CPC with 140 beds, the Clinica Viarnetto with 45 beds, and the Malcantonese Hospital with 26 beds) are located in the southern area of the region, whereas the private clinic of Santa Croce (with 80 beds) is located in the northern area. In addition, the regional network of psychiatric services includes four well-established community mental health teams (eg, sociopsychiatric service [SPS]) available from 9:00 AM to 5:00 PM on weekdays; a service of Psychiatry & Psychological Medicine (SPPM) available from 8:00 AM to 6:00 PM, providing acute psychiatric consultations in five different hospitals; and on-call psychiatrists from both the SPS and the SPPM teams covering psychiatric emergencies from 6:00 PM to 8:00 AM. One of the CPC wards was closed to new inpatient admissions and replaced by the newly established CRHT. Health insurance providers and the Health Department of the Canton of Ticino have agreed to finance CRHT for each patient as if they were treated in an inpatient setting.

### Intervention

The CRHT team is based in Bellinzona and cares for patients aged 18 to 65 years, who would typically be admitted to the CPC on a voluntary basis. The team is available 24 hours a day, 7 days a week (on call from 10:30 PM to 7:00 AM). The new service brings together different health and social care professionals, including 3 physicians (a full-time consultant psychiatrist, a part-time psychiatrist, and a part-time senior consultant psychiatrist on call), 10 mental health nurses, 1 team manager, 1 part-time social worker on call, and 1 part-time clinical psychologist. Referrals are accepted from general practitioners, the local community mental health team, accident and emergency teams, private psychiatrists, and the CPC clinic in Mendrisio. All patients for whom immediate in-patient treatment is deemed necessary have access to the new home treatment service, with the exclusion of people affected by acute alcohol or drug intoxication, extreme agitation, or those who could represent a risk for themselves and others. As referrals may also be accepted by the CPC itself in Mendrisio, patients are considered eligible for the study only if they stayed in the CPC for less than 48 hours before being transferred to the CRHT program. Patients are typically visited at home on a daily basis for approximately 1 h, with the option for multiple visits a day (or night), if necessary. Interventions are individually tailored but include typical components of acute care, such as crisis intervention, pharmacotherapy, psychoeducation, brief psychotherapy, and social care. Key elements addressed by the CRHT include monitoring symptoms; monitoring medication and side effects; identifying and managing safety or risk issues; providing emotional, social, and psychological support; providing carer or family support; liaising with other services and professionals involved in the process of care; and planning discharge meetings and follow-ups. The patient is seen with family members or caregivers from the very beginning, if feasible. This is because the CRHT team provide patients, family members, and carers with elements of psychoeducation on mental health crises along with ways to prevent future relapses and reduce mental illness stigma. In addition, the team promotes an active collaboration with the local SPS, general practitioners, private psychiatrists, and carers to support the long-term needs of those patients.

### Quasi-Experimental Study

#### Overview

The first part of the study evaluates the clinical efficacy of CRHT in Southern Switzerland. In particular, patients aged 18 to 65 years living in two areas in Ticino (Bellinzona e Valli and Lugano) diagnosed with acute mental illness and requiring hospital admission to the CPC were considered for inclusion. Patients at high risk of suicide or self-harm, and those with alcohol or drug problems were excluded from the study. Compulsory admissions were also excluded from the study. Patients in the Bellinzona e Valli area were offered the CRHT service and formed the intervention group; those in the Lugano area formed the control group and received care as usual (ie, hospitalization). Preliminary statistical analysis based on observational data drawn from the CPC database indicates that there are no significant differences in some important health indicators (eg, Brief Symptom Checklist [BSCL] and Health of the Nation Outcome Scales [HoNOS]) between patients living in these two areas. This increases the comparability between the two groups and reduces confounding effects [37]. The study design can, therefore, be considered as a natural experiment based on geography. To calculate the minimal sample size for the study, we used the mean and SD values of the HoNOS scale scores for the experimental and control groups reported in Johnson’s study [15]. To ensure a statistical power of 80%, at the 5% significance level for a 2-tailed hypothesis test, the minimal sample size equals 142. The recruitment period was of 15 months, and every recruited patient was followed for a period of 24 months after discharge.

Quantitative data were collected by the CRHT health care professionals as part of their standard operating procedures for the storage of clinical information, in line with the CPC’s administrative and clinical demands. A team comprising a CPC data manager and researchers from the University of Applied Sciences and Arts of Southern Switzerland (project partner in charge of the data analysis) met on a regular basis in order to monitor the quality of the data collected. The CRHT team checked the eligibility of all patients from both areas of the Canton, according to the abovementioned inclusion criteria. The willingness of patients from Lugano (the northern area) to accept the CRHT was a prerequisite for the intention-to-treat (ITT) analysis, although this theoretical acceptance did not imply any actual assignment to treatment.

#### Outcome Measures

The primary outcome measures of the study are the number of inpatient days; total days in treatment and use of other mental health services; direct costs (treatment and follow-up); and HoNOS and BSCL scores. The secondary outcome measures of the study are patients’ satisfaction (PoC-18 questionnaire); relatives’ satisfaction (PoC-18 questionnaire); occurrence of serious incidents involving deliberate self-harm and violence toward others; satisfaction of the CRHT; and number of days patients were on sick leave and absent from work. Information regarding important heterogeneity factors, such as gender, age, level of education, employment status, unhealthy lifestyle, and the patient’s clinical and social history, including diagnosis and previous service use, are also recorded.

#### Data Analysis

By adopting an ITT approach, the study analyzes all patients who are enrolled regardless of deviations (ie, drop out, protocol deviation, withdrawals, and noncompliance) that may occur after assignment to the treatment and control groups. ITT provides a more reliable estimate of treatment effect, minimizes type-1 errors, and preserves sample size. Univariate tests are used to assess differences between control and treatment groups for all patients’ characteristics, as well as for clinical and nonclinical outcomes. A univariate comparison of questionnaire responders and nonresponders will also be conducted. Following the assumption of Conditional Geographic Treatment Ignorability, we estimate the effects of CRHT on the outcome measures considered by means of generalized linear models and matching techniques, in order to control for some important pretreatment covariates (eg, sociodemographic, clinical, and social variables), with the aim of making treatment and control groups as comparable as possible and adjusting for potential confounders [37-39].

### Qualitative Study

The recruitment phase of the qualitative study started once the observational period of the quantitative study was concluded, in order to avoid potential bias. In particular, the qualitative study aims to investigate the acceptability of the intervention among patients and their carers, as well as among health care professionals of the CRHT team. Further elements to be explored included the interactions between the CRHT team and patients, the role of family members involved by the CRHT team, and the way health care workers collaborated in this new professional context. Data were collected through individual semistructured interviews with a purposeful sampling of patients and their family members and through focus groups with the members of the CRHT team. Two categories of patients were considered for inclusion: (1) those who have accepted the CRHT and have been compliant with it (*per-protocol population*) and (2) those who have accepted the CRHT but have subsequently withdrawn from it (*withdrawn population*). Patients from these two groups had a personal experience with the home treatment service. A comparison between their perspectives is anticipated to provide highlights on the experience of each group and potentially reveals the conditions for successful home treatment. The maximum variation sampling strategy was used in order to maximize the variability of respondents’ experiences [40]. The sample was thus diversified in terms of sex (men or women), age (young or old), family situation (living with family members or not), and psychiatric history (first hospitalization or not), as we anticipated these four characteristics may influence patients’ experience. In line with the pilot study previously conducted [35], we aimed to recruit about 20 dyads (patients and family members) from the *per-protocol population* and about 7 people from the *withdrawn population*. CRHT members were asked to participate in a focus group to explore several aspects, including the challenges of providing such intervention, how the members of the team collaborate within the team, and with the psychiatrist services in Ticino, as well as the forms of collaborations within the team and with the patients and their families. Focus groups were conducted in the premises of the CRHT team by a moderator and an assistant moderator.

Interviews and focus groups were conducted by a researcher not involved in the home treatment team. Data collection and analysis for the interviews were conducted simultaneously, until data saturation was achieved. For this reason, participants were progressively recruited and interviewed based on the themes that emerge from the provisional analysis. All the interviews and the focus groups were audio-recorded, transcribed, and anonymized.

### Economic Evaluation

The last part of the study, for which the data collection is still ongoing, explores the cost-effectiveness of the CRHT intervention implemented. The economic evaluation follows the approach illustrated by McCrone [41]. Direct and indirect costs are obtained for both the treatment and follow-up phases (see Table 1). Treatment costs are provided by the CPC, whereas the follow-up costs are provided by the patients’ health insurance companies. Differences in the health care costs between the two treatments (CRHT vs hospitalization) are assessed using bootstrapped clustered regression analysis. Cost-effectiveness of home treatment are evaluated using cost-effectiveness acceptability curves (CEACs). CEACs involve the treatment and follow-up periods, and these are based on the differences between effectiveness measures and total costs. For the treatment period, the effectiveness measures will include the reduction in the HoNOS and BSCL scores at the end of the treatment, whereas for the follow-up period, the effectiveness measures will include the total number of days without treatment and/or other service utilization and the total number of non-inpatient days registered during the follow-up phase.

**Table 1:** Direct and indirect costs for the intervention and control groups.

Cost type			Intervention group (CRHT^a^)		Control group (hospitalization)
**Direct costs**					
	Treatment phase	Sum of direct medical and nonmedical costs. Direct medical costs include the same variable cost categories as for hospitalized patients (therapies, medication, staff salaries of carers, etc); direct nonmedical costs differ and are mostly attributable to staff travel costs.		Total bed cost per day × total number of inpatient days The total bed cost per day is split into fixed and variable costs. Variable costs include direct medical (therapies, medication, staff salaries of carers, etc) and nonmedical costs (food, accommodation, etc). The fixed cost per bed and day will be calculated by dividing the total fixed cost of the service by the number of inpatient days.	
	Follow-up phase	Direct costs in the follow-up phase correspond to direct medical costs, including the costs of medical consultations, medical emergencies, hospitalizations, pharmaceutical therapies, etc		Same costs for the intervention group	
**Indirect costs**					
	Treatment and follow-up phases	Indirect costs correspond to the costs of lost production. During both phases, the number of days of absence from work will be recorded on the basis of medical certificates issued. The cost of a day of absence from work will be valued using a regional age- and gender-specific average salary.		Same costs for the intervention group	

^a^CRHT: Crisis Resolution and Home Treatment.

### Collaboration

This FORESIGHT (Financial Outputs, Risks, Efficacy, Satisfaction Index and Gate-keeping of Home Treatment in Ticino) study is a joint project of the Organizzazione Sociopsichiatrica Cantonale, the Department of Business Economics, Health and Social Care of the University of Applied Sciences and Arts of Southern Switzerland, and Fondazione Pro Mente Sana. A steering committee that encompasses all important actors (ie, the main applicant, 3 coapplicants, and project partner) will facilitate a continuous interaction between the CRHT team and the researcher team. During the data collection phase, regular meetings will be held in order to monitor the progress of the project.

### Ethics Approval and Consent to Participate

The project is funded by the Swiss National Science Foundation as part of the National Research Program NRP74 (grant 407440_167375). The funding body had no role in the design of the study and collection, analysis, and interpretation of data and in writing the manuscript. The project is approved by the regional Ethics Committee (reference 2017-00247) and is registered as an interventional, nonrandomized, quasi-experimental study (registration ISRCTN38472626). Oral and written information was provided to all patients, and written consent was obtained from all participants.

## Results

The project is funded by the Swiss National Science Foundation as part of the National Research Program NRP74 for a period of 48 months starting from January 2017. As of February 2021, data for the nonrandomized, quasi-experimental study have been collected, and the results are expected to be published by the end of the year. Data are currently being collected and/or analyzed for the qualitative study and economic evaluation. Due to some recruitment issues, the COVID-19 pandemic, and the ensuing substantially limited access to potential participants as well as restrictions for meetings and interviews, it was decided, in accordance with the funding agency, to extend the project until the end of December 2021. The updated schedule of the project is presented in a table in Multimedia Appendix 1.

## Discussion

### Principal Findings

This paper describes the protocol for a mixed methods study designed to assess the clinical efficacy, acceptability, and cost-effectiveness of a new home treatment intervention for people affected by acute psychiatric crises. Compared to other Swiss CRHT experiences, the CRHT service in Ticino represents a unique case, as the introduction of the service is backed by the closing of one of its acute wards. Therefore, this home treatment experience has the specific characteristic of being addressed to all patients, with an acute psychiatric crisis living in the northern area of Ticino and eligible for treatment at home, rather than a selected subgroup of patients.

Crisis care for service users, where support is provided during a crisis either in their home or in a community setting, is found by several reviews to provide a package of support that is worthwhile, acceptable, and less expensive than standard care [26,42-44]. In particular, crisis care has the potential to avoid repeated admissions to hospital and improve the mental state of services users more than standard care among this group. To increase the chances of a successful implementation, the CRHT intervention has been planned and designed together with local health professionals and the support of the relevant stakeholders in the Canton.

To our knowledge, this is the first study to implement and evaluate a CRHT intervention in Southern Switzerland. The choice of conducting a mixed methods study, which involves a quasi-experimental design, a qualitative study, and a cost-effectiveness analysis, is supported by the idea of evaluating the CRHT intervention comprehensively and from different perspectives thanks to the input of a multidisciplinary team. In addition to gathering preliminary data on the efficacy of this program for improving health-related outcomes among the target group, the proposed study also gathers valuable data on program engagement and experiences in the program among the target group and their carers. This is important given that only a small number of studies have investigated patients’ and carers’ experiences in relation to CRHT services. Conducting interviews with participants (potentially including those who drop out of the program) will allow the researchers to gain insight into how people approach the service and live the home visits conducted by the CRHT team. The proposed study will integrate a cost-effectiveness analysis to determine the incremental cost-effect of the program compared to treatment as usual. This will deliver a preliminary understanding of whether the program provides value for money compared with hospitalization.

Given this is the first experience of home treatment in Southern Switzerland, it is anticipated that the findings from this study will potentially have an extensive impact at the local level. In particular, these findings will inform the refinement and extended implementation of CRHT intervention in other areas of the Canton, as well as the development of ad hoc educational interventions to train health care professionals, including nurses and doctors, on crisis interventions and home treatment services. In addition, thanks to the multitude of data collected, the research team will be able to draw further recommendation on CRHT service in terms of clinical efficacy, as well as patients and providers’ acceptability and cost-effectiveness compared to the standard inpatient treatment.

### Conclusions

The FORESIGHT study aims to address several topics related to the home treatment of acute mental crisis for which there is no evidence or consistent findings, specifically, whether the CRHT service provided in Ticino is clinically effective, the determinants of its feasibility and acceptability, and the satisfaction of those that receive and those that provide the service. This study also has the potential to extend our current theoretical understanding of the mechanisms of action underlying home treatment interventions for people affected by an acute psychiatric crisis. Finally, the study will identify important results in relation to the CRHT service delivery and its cost-effectiveness as an alternative to hospitalization for crisis resolution. Establishing the feasibility and effectiveness of the CRHT in Ticino could provide a scalable solution for improving the mental health and quality of life of people with mental disorders.

## Appendix

Multimedia Appendix 1 Project plan: 5-year timeline.

## Abbreviations

BSCL: Brief Symptom Checklist
CEAC: Cost-Effectiveness Acceptability Curve
CPC: Cantonal Psychiatric Clinic
CRHT: Crisis Resolution and Home Treatment
HoNOS: Health of the Nation Outcome Scales
ITT: intention to treat
RCT: randomized controlled trial
SPPM: Service of Psychiatry and Psychological Medicine
SPS: sociopsychiatric service
